# Enhanced dsRNA Production via a Three-Terminator Vector and Transcriptomic Correlates of RNAi Exposure in Thrips

**DOI:** 10.3390/insects17070685

**Published:** 2026-07-01

**Authors:** Lin Tian, Guangtao Xu, Jianyu Li, Yixuan Zhang, Wei Shang, Junhua Xie, Yucheng Gu, Yanna Huang, Xueming Tang

**Affiliations:** 1 School of Agriculture and Biology, Shanghai Jiao Tong University, Shanghai 200240, China; sjtutianlin@sjtu.edu.cn (L.T.);; 2 Key Laboratory of Urban Agriculture, Ministry of Agriculture and Rural Affairs, Shanghai 201403, China; 3 Shanghai Yangtze River Delta Eco-Environmental Change and Management Observation and Research Station (Shanghai Urban Ecosystem Research Station), Ministry of Science and Technology, National Forestry and Grassland Administration, Shanghai 201602, China; 4 Handan Hangang Hospital, Handan 056001, China; 5 Stockbridge School of Agriculture, University of Massachusetts, Amherst, MA 01003, USA; 6 Faculty of Life Sciences, University College London, London WC1E 6BT, UK; 7 School of Biotechnology, East China University of Science and Technology, Shanghai 200237, China; 8 Syngenta Jealott’s Hill International Research Centre, Bracknell, Berkshire RG42 6EY, UK; 9 Key Laboratory of RNA Biopesticide Technology, Ministry of Agriculture and Rural Affairs, Shanghai 201314, China

**Keywords:** RNAi, dsRNA, clathrin-mediated endocytosis, SID-1-like protein, *Megalurothrips usitatus*, *Frankliniella occidentalis*

## Abstract

RNA interference (RNAi) represents a promising alternative to chemical insecticides, but its efficacy depends on efficient double-stranded RNA (dsRNA) uptake, a process poorly characterized in thrips. To enable sufficient dsRNA production for functional studies, we first optimized an *E. coli* expression system by constructing a vector containing three tandem terminators, which substantially enhanced dsRNA yield by approximately 11-fold. Using this optimized production system, this study identified a conserved *muscle actin* fragment for dsRNA synthesis and evaluated RNAi responses in *Megalurothrips usitatus* and *Frankliniella occidentalis*. Insect mortality, target-gene suppression, and transcriptomic responses were evaluated via RT-qPCR and RNA-seq analyses using artificial diets supplemented with *muscle actin* dsRNA. The designed ds*actin* shared > 97% sequence identity between the two species. Oral ingestion of 1500 ng µL^−1^ dsRNA caused concentration-dependent mortality (72% in *M. usitatus*, 48% in *F. occidentalis*) and significant down-regulation of *muscle actin* mRNA within 72 h. Transcriptomic analysis in *M. usitatus* revealed upregulation of genes associated with clathrin-mediated endocytosis and SID-1-like transmembrane transport, suggesting a potential dual-pathway model for dsRNA uptake. These findings provide correlative insights into RNAi efficiency in thrips.

## Simple Summary

Thrips are tiny but devastating agricultural pests, causing significant crop damage and economic losses globally. Current reliance on chemical pesticides leads to problems like pest resistance, environmental harm, and health concerns. RNA interference, a natural process that can silence specific genes, offers a greener pest control method. However, its widespread use is hindered by the high cost of producing the necessary double-stranded RNA molecules and a limited understanding of how insect cells take up these molecules. In our research, we significantly boosted double-stranded RNA production by optimizing a bacterial system with three tandem terminators. We then tested the insecticidal effect of double-stranded RNA targeting a highly conserved *muscle actin* gene on two major thrips species, *Megalurothrips usitatus* and *Frankliniella occidentalis*. Our results showed that oral delivery of the designed double-stranded RNA caused significant mortality and target gene suppression in both species, and gene expression analysis identified two cellular pathways likely involved in double-stranded RNA uptake. These findings provide technical and theoretical support for developing sustainable RNA biopesticides against thrips, helping to reduce agricultural dependence on chemical insecticides.

## 1. Introduction

RNA interference (RNAi) is an evolutionarily conserved, sequence-specific post-transcriptional gene silencing mechanism that has emerged as a promising molecular strategy for controlling insect pests [1,2]. This process is initiated by the introduction of double-stranded RNA (dsRNA) that is homologous to a target messenger RNA (mRNA), leading to the formation of small interfering RNAs (siRNAs) through Dicer-2 endonuclease cleavage [3,4]. These siRNAs are subsequently incorporated into the RNA-induced silencing complex (RISC), guiding Argonaute-mediated degradation of complementary mRNA and ultimately suppressing gene expression [5,6]. In insects, this mechanism can be exploited for pest management through the ingestion or topical application of dsRNA; however, RNAi efficacy varies widely among taxa and is strongly constrained by how dsRNA is internalized and transported at the cellular level [7,8].

A critical prerequisite for RNAi-based applications is the availability of sufficient quantities of high-quality dsRNA. Conventional in vitro synthesis methods are often cost-prohibitive for large-scale applications, whereas bacterial production systems offer a scalable and economical alternative. In *Escherichia coli*, dsRNA expression is typically driven by T7 RNA polymerase (T7 RNAP), but transcription efficiency is frequently limited by inefficient transcription termination, leading to heterogeneous transcripts and reduced yields. The use of multiple tandem terminators has been shown to enhance transcription termination efficiency and increase dsRNA yield [9,10]. Therefore, optimizing dsRNA production through a vector using multiple transcriptional terminators represents a key step toward enabling robust RNAi applications.

The bean flower thrips (*Megalurothrips usitatus*) and the western flower thrips (*Frankliniella occidentalis*) are among the most destructive agricultural pests worldwide, attacking a wide range of vegetable and ornamental crops [11,12]. Both species damage plants directly by feeding on host mesophyll cells and indirectly by transmitting plant viruses [13,14]. Due to their short life cycle, parthenogenetic reproduction, and cryptic behavior, thrips rapidly develop resistance to multiple classes of chemical insecticides [12,15,16]. Consequently, new molecular control strategies are urgently needed. RNAi-based pest control offers an alternative that circumvents conventional resistance mechanisms and may complement integrated pest management (IPM) programs [17].

Previous studies have demonstrated the feasibility of RNAi in *F. occidentalis* using various delivery methods and target genes [18,19,20,21], but the cellular uptake mechanisms remain largely unexplored in Thysanoptera. Actin, a highly conserved protein, polymerizes to form filaments that assemble into cross-linked networks within the cellular cytoplasm. In insects, muscle actin is a major actin isoform predominantly expressed in muscle tissues. Wu et al. used transgenic tobacco plants expressing dsRNA targeting *β-actin* of *F. occidentalis*, resulting in high mortality [18]. Transgenic technology expressing dsRNA offers an efficient control method for thrips, but it raises public safety concerns. Compared with the ubiquitous *β-actin*, *muscle actin* represents a potentially safer and equally lethal target. Furthermore, field infestations of thrips consistently involve multispecies (e.g., *M. usitatus* and *F. occidentalis*). Thus, the development of dsRNA targeting the highly conserved *muscle actin* gene represents a potentially promising strategy for thrips control.

The success of RNAi in insects largely depends on the efficient cellular uptake and systemic transport of dsRNA. Two principal pathways have been described for dsRNA internalization: (1) SID-1-dependent transmembrane transport, first characterized in *Caenorhabditis elegans* [22,23] and later found in some insects or mammals such as SID-1-like proteins [24,25,26,27]; and (2) clathrin-mediated endocytosis (CME), a vesicular uptake route demonstrated in several insect orders including Coleoptera [28], Hemiptera [29], and Orthoptera [30]. However, the presence and relative contributions of these uptake mechanisms can vary substantially among insect taxa, often determining whether RNAi is robust or refractory. To date, the cellular basis of dsRNA uptake has not been elucidated in Thysanoptera, leaving a critical gap in understanding why RNAi efficiency differs across thrips species.

In the present study, we first optimized dsRNA production by constructing a bacterial expression vector carrying three tandem terminators, which significantly enhanced dsRNA yield. Using this optimized production system, we aimed to elucidate the basis of dsRNA uptake in Thysanoptera, a taxonomic group for which the mechanisms of RNAi remain largely unknown. Specifically, we (1) evaluated the potential of a highly conserved *muscle actin* gene as a cross-species lethal target for RNAi in *M. usitatus* and *F. occidentalis* through feeding bioassays; (2) determined whether dsRNA uptake in thrips is associated with SID-1-like transmembrane transport and/or clathrin-mediated endocytosis by analyzing transcriptomic responses; and (3) characterized the transcriptional responses and functional pathways activated following dsRNA exposure to establish a correlative framework for RNAi efficacy in thrips. By addressing these objectives, this work filled a key knowledge gap in the biochemical understanding of RNAi uptake in Thysanoptera and laid the groundwork for developing RNAi-based biopesticides with improved delivery efficiency and cross-species activity.

## 2. Materials and Methods

### 2.1. Insect Strains and Rearing Conditions

Colonies of *M. usitatus* and *F. occidentalis* were originally collected from a soybean field in Shanghai, China (31.0773° N, 121.5115° E), in May 2024. Populations were maintained on fresh bean pods under controlled laboratory conditions (26 ± 2 °C, 50 ± 10% relative humidity, and 16:8 h light: dark photoperiod). *F. occidentalis* can be reared in trays enclosed with 80-mesh net cages, whereas *M. usitatus*, owing to its strong escape tendency, was kept in ventilated containers fitted with 120-mesh stainless steel screens. Species identity was confirmed morphologically and further validated by nucleotide sequencing: the mitochondrial cytochrome oxidase subunit I (COI) region was used for *M. usitatus*, and the internal transcribed spacer 2 (ITS2) region was used for *F. occidentalis*. Amplifications were performed with published primers LCOI1490/HC2198 [31] and CAS5p8sFc/CAS28sB1d [32] (Table S1).

### 2.2. Target Gene Selection and Sequence Analysis

The *muscle actin* gene, a conserved structural protein predominantly expressed in insect muscle tissues, was selected as the RNAi target due to its high sequence conservation and functional indispensability. Full-length *muscle actin* sequences from *M. usitatus* (OR711914.1) and *F. occidentalis* (XM_052268144.1) were aligned, and a 400 bp conserved region (nucleotides 549–948 and 520–920, respectively) was identified for dsRNA synthesis using an in-house computational algorithm (Table S2). This 400 bp fragment was used to synthesize a cross-species dsRNA molecule. To clearly distinguish it from a species-specific *muscle actin* dsRNA, this molecule is hereafter referred to as ds*actin*.

The phylogeny of *muscle actin* was constructed based on the corresponding *muscle actin* gene sequences of representative insect species from different orders. GenBank accession numbers of *muscle actin* from other thrips species and insect orders were obtained from NCBI (Table S3). Sequence alignment was performed using Clustal W as implemented in MEGA X. A phylogenetic tree was constructed using the Neighbor-Joining method with 1000 bootstrap replicates, employing pairwise deletion for gap treatment. The resulting tree was visualized and annotated using iTOL (https://itol.embl.de/, accessed on 15 October 2025) [33].

### 2.3. In Vitro dsRNA Synthesis

Template DNA fragments containing T7 promoter sequences at both ends were synthesized by Genewiz (Suzhou, Jiangsu Province, China). dsRNA was synthesized using the T7 RNAP system (Silicon Gene Technologies, Shanghai, China) at 37 °C for 2 h in a total reaction volume of 500 µL containing NTPs and reaction buffer. To visualize ingestion, Cy3-UTP (Beyotime, Shanghai, China) was substituted for 35% of the standard UTP in selected reactions. Residual DNA and proteins were removed by sequential digestion with DNase I (37 °C, 1 h; Beyotime, Shanghai, China) and Proteinase K (37 °C, 30 min; Beyotime, Shanghai, China), followed by TRIzol/chloroform extraction (Beyotime, Shanghai, China) and ethanol precipitation, and then finally purified with RNA Clean Beads (Vazyme, Nanjing, Jiangsu Province, China). dsRNA purity and concentration were confirmed using a Nanodrop 2000 spectrophotometer (Thermo Fisher Scientific, Waltham, MA, USA).

### 2.4. Optimization of dsRNA Production in Escherichia coli

#### 2.4.1. Plasmid Construct Design

The pET28a vector and the *E. coli* HT115(DE3) strain were purchased from Weidi Biotechnology Co., Ltd. (Shanghai, China). Synthetic DNA fragments were procured from Genewiz (Suzhou, Jiangsu Province, China) and subsequently inserted into the pET28a vector via homologous recombination using Exnase II (Vazyme, Nanjing, Jiangsu Province, China) or restriction enzyme digestion using restriction enzymes (New England Biolabs, Ipswich, MA, USA) and T4 DNA Ligase (New England Biolabs, Ipswich, MA, USA). tT7 [9] and rrnB T1 [10] represent a class I T7 synthetic terminator and the multiclass endogenous *E. coli* terminator, respectively (Table S2). Three pET28a-based plasmid constructs were designed to express dsRNA targeting the *muscle actin* gene: no terminator (0 Ter), a single T7 terminator (1 Ter), and three tandem terminators (3 Ter). All constructs contain two convergent T7 promoters flanking a cloning region. The 3 Ter construct includes three terminators (tT7 + rrnB T1 + tT7) on each side of the expression cassette. Detailed cloning strategies are provided in Supplementary Methods.

#### 2.4.2. *E. coli* Cell Growth and Inductions

Three individual colonies that were confirmed by Sanger sequencing (Sangon, Shanghai, China) to contain the correct insert sequence and the correct terminator configuration were used as biological replicates. From each verified monoclonal bacterial culture, 10 µL was inoculated into 5 mL of LB medium (containing 50 µg mL^−1^ kanamycin) in a 15 mL tube and grown overnight at 37 °C with shaking at 200 rpm. After overnight incubation, 3 mL of the *E. coli* cells was transferred into 60 mL of fresh LB medium (containing 50 µg mL^−1^ kanamycin) in a 250 mL flask and incubated at 37 °C, 200 rpm for 2 h until the OD_600_ reached 0.6–0.8. Induction was initiated by the addition of IPTG (Sangon, Shanghai, China) to a final concentration of 0.5 mM, and the cells were then incubated for an additional 8 h at 37 °C, 200 rpm. Cell growth was monitored by measuring OD600 at −2 h and −1 h (before induction) and at 0, 2, 4, 6, and 8 h post-induction using a NanoDrop 2000 spectrophotometer.

#### 2.4.3. dsRNA Extraction, Purification, and Quality Assessment

Cells were aliquoted at 30 × 10^9^ cells, calculated using the online tool provided by Bio Calculators (https://www.agilent.com.cn/store/biocalculators/calcODBacterial.jsp, accessed on 30 September 2024). Cells from the culture were harvested by centrifugation at 6000 rpm for 5 min at 4 °C. dsRNA was extracted using TRIzol reagent (Beyotime, Shanghai, China) via phenol-chloroform extraction and isopropanol (Sangon, Shanghai, China) precipitation, treated with DNase I and RNase T1, re-extracted with phenol-chloroform, and then finally purified with RNA Clean Beads (Vazyme, Nanjing, Jiangsu Province, China). The RNA concentration was determined using a Nanodrop 2000 spectrophotometer. To verify dsRNA integrity and size, 1% agarose gel electrophoresis was performed. The intensity of dsRNA bands on agarose gels was quantified using ImageJ 2.0 software (National Institutes of Health, Bethesda, MD, USA). Briefly, gel images were imported into ImageJ, converted to 8-bit grayscale, and the background was subtracted. A rectangular region of interest (ROI) was drawn around each target band, and the integrated density (sum of pixel values within the ROI) was measured. Relative band intensity was calculated as the integrated density of each band, with background signal subtracted, and used for subsequent quantitative analysis.

### 2.5. RNAi Bioassay via Membrane Feeding

Feeding bioassays were performed following Singh et al. [34] with minor modifications. Artificial diets consisted of 1% (*w*/*v*) sucrose (Sangon, Shanghai, China) and 25 mg mL^−1^ heat-treated pine pollen (Tongrentang, Beijing, China) sandwiched between two layers of Parafilm. dsRNA was mixed into the diet at 300–1500 ng µL^−1^, and 30 adults were confined per 30 mm feeding chamber. To assess stability, ds*actin* at both the lowest (300 ng µL^−1^) and highest (1500 ng µL^−1^) test concentrations was incorporated into the Parafilm-sandwiched artificial diet and sampled at 0, 4, 8, 12, 24, 48, and 72 h. Electrophoretic analysis on a 1% agarose gel confirmed that the dsRNA remained intact at all time points (Figure S1). Mortality was recorded over 72 h post-feeding. To confirm dsRNA ingestion and rule out autofluorescence, thrips were fed Cy3-labeled *eGFP* dsRNA (ds*eGFP*) and compared with non-labeled ds*eGFP*-fed controls. dsRNA ingestion was verified using a confocal laser scanning microscope (Nikon Ti2, Tokyo, Japan) after a 24 h feeding period with 100 ng µL^−1^ Cy3-labeled ds*eGFP* in 30 adults each of *M. usitatus* and *F. occidentalis*. For confocal imaging, Cy3 fluorescence (excitation 561 nm, emission 570–616 nm) was detected with a gain setting of 44.0, while transmitted light (TD) images were captured at a detector gain of 37.0. Fluorescence intensity was quantified using ImageJ software. For each adult sample, the raw integrated density was measured.

### 2.6. RNA Extraction and Quantitative Real-Time PCR (RT-qPCR)

Total RNA was extracted from pools of adult thrips (30 individuals per biological replicate) using a commercial RNA isolation kit (Vazyme, Cat. No. RC112, Nanjing, Jiangsu Province, China). RNA integrity was confirmed by 1% agarose gel electrophoresis and quantified using a NanoDrop 2000 spectrophotometer. First-strand cDNA was synthesized from 150 ng total RNA with HiScript III RT SuperMix (+gDNA wiper) (Vazyme, Nanjing, Jiangsu Province, China). RT-qPCR was conducted using ChamQ Blue Universal SYBR qPCR Master Mix (Vazyme, Nanjing, Jiangsu Province, China) on SLAN-96S real-time PCR system (Hongshi Medical Technology Co., Ltd., Shanghai, China). Reference genes were *gapdh* [35] for *M. usitatus* and *tubulin* [36] for *F. occidentalis*. Primer efficiency (95–105%) and single-peak melting curves were verified before quantification. Relative gene expression levels were calculated using the 2^−ΔΔCt^ method [37], based on three to six biological replicates, each with three technical replicates. The 24 h ds*eGFP*-treated group was designated as the reference sample for both *M. usitatus* and *F. occidentalis*.

### 2.7. Transcriptome Sequencing and Analysis

Adult *M. usitatus* were fed diets containing 300 ng µL^−1^ ds*actin*, 300 ng µL^−1^ ds*eGFP*, or ddH_2_O for 72 h, with three biological replicates per treatment (30 individuals per replicate). A sublethal concentration (300 ng µL^−1^) was selected for transcriptomic analysis to minimize secondary transcriptional effects associated with advanced morbidity and mortality observed at higher dsRNA concentrations. Total RNA (≥1 µg; RQN > 6.5) was extracted and used for library preparation after poly(A) mRNA enrichment with oligo(dT) beads. Fragmented mRNA (~300 bp) was converted to cDNA, end-repaired, A-tailed, and ligated with sequencing adapters. Libraries were size-selected, PCR-amplified, and sequenced (2 × 150 bp) on an Illumina NovaSeq X Plus platform. Clean reads were mapped to the *M. usitatus* reference genome (NCBI Assembly GCA_029381295.1) using HISAT2. Differentially expressed genes (DEGs) were identified using DESeq2 (version 1.42.0) (*p*-adjust < 0.05, |log_2_^FC^| ≥ 2). Functional annotation and pathway enrichment analyses were performed against KEGG and GO databases.

### 2.8. Validation of Transcriptome Data

Twelve DEGs associated with RNA transport, endocytosis, and stress response were selected for RT-qPCR validation based on the following criteria: (1) a balanced range of fold changes (|log_2_^FC^| between 0 and 2 and between 2 and 4); and (2) differential expression across at least two of the three comparisons (ds*actin* vs. ds*eGFP*, ds*actin* vs. ddH_2_O, ds*eGFP* vs. ddH_2_O). Gene-specific primers were listed in Table S1. Spearman correlation test was used to evaluate the correlation between RNA-seq and RT-qPCR gene expression data.

### 2.9. Statistical Analysis

Statistical analyses and visualization were performed using GraphPad Prism 8.0. Transcriptomic data were analyzed and visualized with the proprietary bioinformatics pipeline provided by Majorbio Bio-pharm Technology Co., Ltd. (Shanghai, China). The dot plot was generated using Python 3.11. Mortality data were subjected to Tukey’s multiple comparisons test. Gene expression differences were evaluated using multiple *t*-tests with FDR correction (two-stage step-up method of Benjamini, Krieger and Yekutieli) in GraphPad Prism. An FDR < 0.01 was considered statistically significant. Significance was accepted at *p* < 0.05. All schematic figures presented in the Results section were prepared with BioRender (https://biorender.com, accessed on 16 March 2026).

## 3. Results

### 3.1. Efficient dsRNA Production via a Three-Terminator Vector for Feeding Bioassays

**Figure 1:**
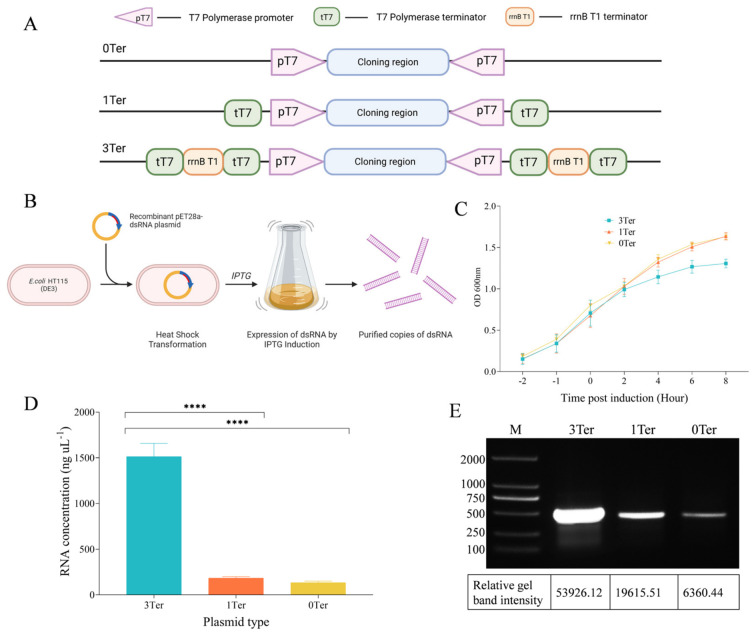
Enhanced dsRNA production using a three-terminator expression system in *E. coli*. (**A**) Schematic illustration of the plasmid constructs designed for dsRNA synthesis using transcriptional terminators. The cloning region was inserted into ds*actin* or ds*eGFP*. The various T7 terminators used are indicated in the key. (**B**) Schematic illustration of dsRNA production in the *E. coli* expression system. Recombinant pET28a-dsRNA was transformed into *E. coli* HT115(DE3), and subsequently IPTG was added to induce dsRNA expression. Finally, purified dsRNA was extracted. (**C**) Growth curve comparison of *E. coli* HT115(DE3) cells transformed with each of the plasmids. An outgrowth was performed and allowed to grow to an OD_600_ of 0.6–0.8. Samples were then induced for 8 h, with OD measurements recorded at hourly time points. Data are shown as mean ± SD and are representative of three biological replicates. (**D**) RNA concentration per 30 × 10^9^ cells measured by Nanodrop 2000. The RNA was eluted in 100 µL. Data are shown as mean ± SD and are representative of three biological replicates. (**E**) 1% Agarose gel electrophoresis of extracted ds*actin* from different terminator constructs (400 bp). For each lane, 1 µL of sample was loaded, and 5 µL of DL2000 DNA marker was used. Statistical significance was determined using Student’s *t*-test: **** *p* < 0.0001.

To support subsequent feeding assays, we first optimized dsRNA production in *E. coli* using the pET28a vector containing three tandem terminators (Figure 1A,B). We selected two well-characterized rho-independent terminators, tT7 and rrnB T1, which function efficiently with T7 RNAP [10]. The pET28a vector was engineered with two convergent T7 RNAP promoters flanking a target dsRNA sequence, combined with different terminator configurations: no terminator (0 Ter), a single T7 terminator (tT7; 1 Ter), and a combination of tT7 + rrnB T1 + tT7 (3 Ter) (Figure 1A). Recombinant pET28a-dsRNA plasmids were transformed into *E. coli* HT115(DE3) cells, and dsRNA was extracted following IPTG induction (Figure 1B).

Cell growth was monitored by measuring OD_600_. At 4, 6, and 8 h post-induction, the cell count in the 3 Ter group was significantly lower than that in the 0 Ter and 1 Ter groups (Figure 1C; Student’s *t*-test: at 4 h, 3 Ter vs. 1 Ter, *p* = 0.0014, 3 Ter vs. 0 Ter, *p* = 0.0045; at 6 h, 3 Ter vs. 1 Ter, *p* < 0.0001, 3 Ter vs. 0 Ter, *p* = 0.0008; at 8 h, 3 Ter vs. 1 Ter, *p* < 0.0001, 3 Ter vs. 0 Ter, *p* < 0.0001). We next evaluated the RNA concentration per 30 × 10^9^ cells for each plasmid type, using a uniform elution volume of 100 µL. The dsRNA level in the 3 Ter group was significantly higher than that in the 1 Ter group (Student’s *t*-test, *p* < 0.0001) and the 0 Ter group (Student’s *t*-test, *p* < 0.0001). The mean RNA concentration in the 3 Ter group was approximately 11.37-fold higher than that in the 0 Ter group. Agarose gel electrophoresis confirmed the successful synthesis of ds*actin* (400 bp) for all plasmid constructs, and semi-quantitative analysis of band intensity indicated that the 3 Ter plasmid yielded the highest dsRNA levels (Figure 1E). Collectively, these results indicate that the inclusion of three terminators substantially increased dsRNA production, achieving an 11.37-fold enhancement in yield.

### 3.2. Phylogenetic Analysis and Sequence Conservation of the Muscle Actin Gene

**Figure 2:**
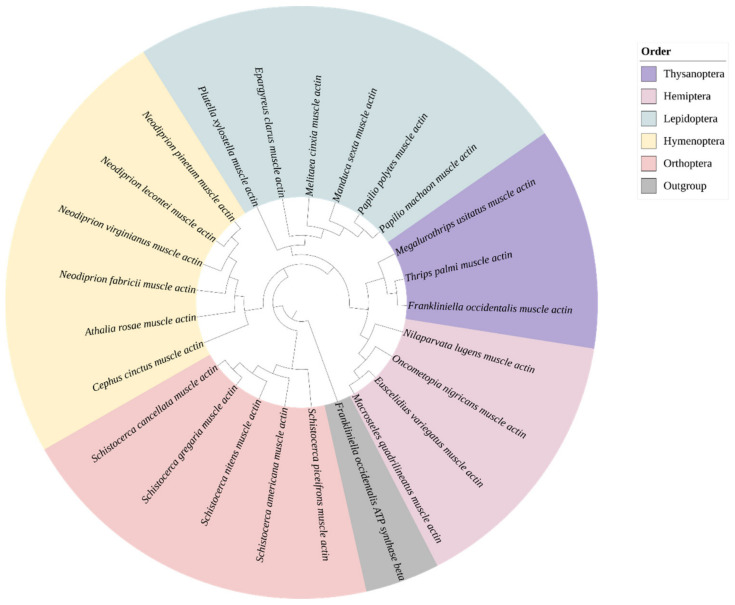
Phylogenetic tree of *muscle actin* sequences from different insect orders. The tree was constructed using the Neighbor-Joining method based on full-length mRNA sequences of representative insect species from various orders. *F. occidentalis ATP synthase beta* mRNA sequence was used as an outgroup. Nucleotide sequences were retrieved from NCBI. The tree was visualized using iTOL v7 software.

To confirm that the targeted fragment is specific to the *muscle actin* isoform and to assess its conservation across Thysanoptera, we performed phylogenetic analysis as described below. Phylogenetic reconstruction based on *muscle actin* sequences from representative insect orders produced four distinct clusters (Figure 2). The *muscle actin* genes of *M. usitatus* and *F. occidentalis* grouped closely within the Thysanoptera clade, indicating strong evolutionary conservation. The Thysanoptera cluster showed a closer relationship to Hemiptera and Lepidoptera compared with Hymenoptera and Orthoptera. Alignment of *muscle actin* gene sequences from *M. usitatus* and *F. occidentalis* revealed high nucleotide identity within the selected 400 bp target region used for dsRNA synthesis—97.0% for *M. usitatus* and 98.3% for *F. occidentalis* (Figure S2).

### 3.3. Oral Ingestion and RNAi Effects of Dsactin in Thrips

**Figure 3:**
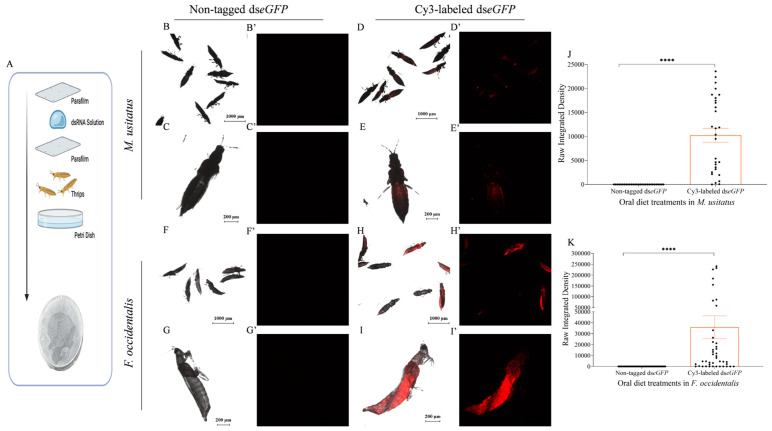
Membrane feeding assay for RNAi in thrips. (**A**) Schematic illustration of the membrane feeding method. (**B**–**I**) Representative confocal laser scanning microscopy (CLSM) images of adult thrips after feeding on an artificial diet containing 100 ng µL^−1^ non-tagged or Cy3-labeled ds*eGFP*. *M. usitatus* adults (n = 30) were fed with non-tagged ds*eGFP* (**B**,**C**) or Cy3-labeled ds*eGFP* (**D**,**E**). *F. occidentalis* adults (n = 30) were fed with non-tagged ds*eGFP* (**F**,**G**) or Cy3-labeled ds*eGFP* (**H**,**I**). Merged images of fluorescence and brightfield channels are shown in (**B**–**I**), and the corresponding fluorescence signals alone are presented in (**B’**–**I’**). Quantification of fluorescence intensity (Raw Integrated Density) in *M. usitatus* (**J**) and *F. occidentalis* (**K**) fed with non-tagged ds*eGFP* or Cy3-labeled ds*eGFP*. The raw integrated density was measured using ImageJ as an indicator of the total uptake of Cy3-labeled dsRNA, with 30 biological replicates included per treatment. Statistical analysis via Student’s *t*-test showed that the fluorescence signal in the Cy3-labeled ds*eGFP* groups was significantly higher than that in the non-tagged ds*eGFP* control groups for both species (**** *p* < 0.0001). Error bars represent mean ± SEM. Scale bars: 1000 µm for images showing multiple individuals (**B**,**D**,**F**,**H**), and 200 µm for single-individual close-up images (**C**,**E**,**G**,**I**).

To verify dsRNA ingestion, a membrane-feeding assay was established, as illustrated in Figure 3A. In *M. usitatus*, red fluorescence was observed in adults fed with Cy3-labeled ds*eGFP* (Figure 3D,D’,E,E’). In *F. occidentalis*, adults fed under the same conditions also exhibited red fluorescence, which was localized in the digestive tract, including the oral cavity and abdominal region (Figure 3H,H’,I,I’). No fluorescence was detected in individuals of either species fed with non-tagged ds*eGFP* (Figure 3B,B’,C,C’ for *M. usitatus*; Figure 3F,F’,G,G’ for *F. occidentalis*), confirming that the observed signal was not due to autofluorescence. Quantification of fluorescence intensity using ImageJ revealed a significantly higher signal in both species fed with Cy3-labeled ds*eGFP* compared to the non-tagged ds*eGFP* controls (Student’s *t*-test, *p* < 0.0001; Figure 3J,K). Collectively, these data verify successful oral uptake of dsRNA by both thrips species in the artificial membrane feeding bioassay.

**Figure 4:**
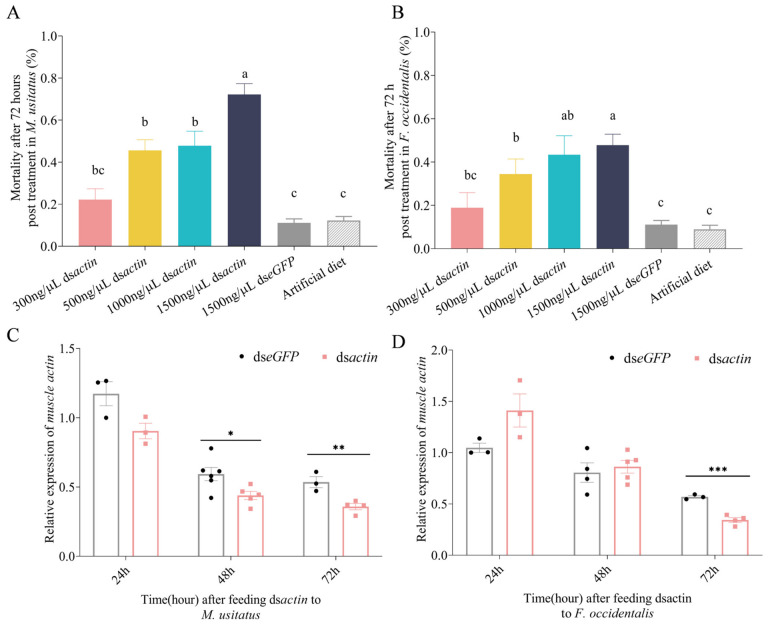
Effects of ds*actin* feeding on adult mortality and target gene expression in two thrips species. (**A**,**B**) Mortality of *M. usitatus* (**A**) and *F. occidentalis* (**B**) over 72 h following continuous feeding on artificial diets containing increasing concentrations of ds*actin* (300–1500 ng µL^−1^). Control groups were fed with ds*eGFP* or ddH_2_O. Different letters above the bars indicate significant differences among treatments at the same time point (*p* < 0.05). (**C**,**D**) Time-course relative expression of *muscle actin* in *M. usitatus* (**C**) and *F. occidentalis* (**D**) after feeding with 300 ng µL^−1^ ds*actin* or ds*eGFP*, measured by RT-qPCR. Relative expression was measured at 24, 48, and 72 h post-treatment. Error bars represent mean ± SEM. Mortality data were analyzed using Tukey’s multiple comparisons test. Statistical significance in RT-qPCR was determined using multiple *t*-tests with FDR correction: * *p* < 0.05, ** *p* < 0.01, *** *p* < 0.001.

Feeding bioassays with increasing concentrations of ds*actin* (300–1500 ng µL^−1^) resulted in a concentration-dependent increase in adult mortality in both thrip species within 72 h (Figure 4A,B). At the highest concentration tested (1500 ng µL^−1^), mortality in *M. usitatus* reached 72.2% ± 5.1%, whereas *F. occidentalis* exhibited a mean mortality of 47.8% ± 5.1%. Mortality in the ds*eGFP* and ddH_2_O control groups stayed at persistently low levels throughout the entire experimental period.

To assess gene silencing efficiency, relative *muscle actin* transcript abundance was measured by RT-qPCR following feeding with 300 ng µL^−1^ ds*actin* (Figure 4C,D). In *M. usitatus*, *muscle actin* expression was reduced by 26.0% at 48 h (*p* = 0.0274) and by 33.1% at 72 h (*p* = 0.0092) compared with the ds*eGFP* control. In *F. occidentalis*, transcript levels were reduced by 39.6% at 72 h (*p* = 0.0007). Collectively, these results demonstrate that ingestion of ds*actin* via the membrane feeding assay led to significant *muscle actin* gene silencing and increased adult mortality in both thrips species within the 72 h period under the tested conditions.

### 3.4. Transcriptomic Responses to Dsactin Feeding in M. usitatus

RNA-seq analysis was performed to characterize transcriptional changes in adult *M. usitatus* following 72 h of feeding on diets containing 300 ng µL^−1^ ds*actin*, ds*eGFP*, or ddH_2_O. Sequencing generated high-quality data with Q20 > 98% and Q30 > 95%. Read-mapping rates ranged from 70.3% to 83.6% against the *M. usitatus* reference genome, indicating adequate coverage for differential expression analysis.

**Figure 5:**
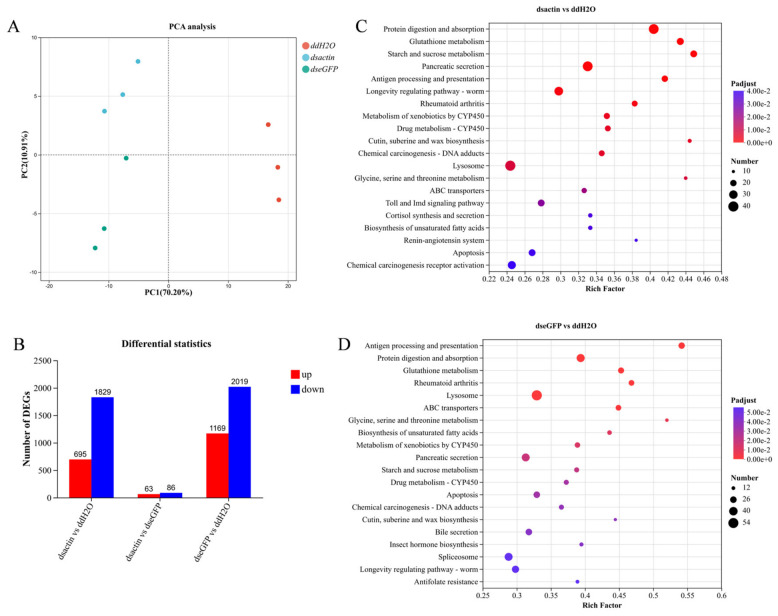
PCA, differential statistics, and KEGG pathways according to RNA-seq data. (**A**) PCA among three biological repeats in three treatments: ds*actin*, ds*eGFP*, and ddH_2_O. (**B**) DEGs between three treatments: ds*actin*, ds*eGFP*, and ddH_2_O. (**C**,**D**) KEGG enrichment analysis between ds*actin* and ddH_2_O (**C**), between ds*eGFP* and ddH_2_O (**D**).

Principal component analysis (PCA) showed distinct clustering among the three treatments (Figure 5A), confirming reproducible transcriptional differences among biological replicates. Relative to the ds*eGFP* treatment, feeding on ds*actin* resulted in 86 down-regulated and 63 up-regulated genes. When compared with the ddH_2_O control, 1829 genes were down-regulated, and 695 genes were up-regulated (Figure 5B). Functional enrichment based on KEGG annotation showed that DEGs were primarily associated with pathways including protein digestion and absorption, lysosome, and endocytosis (Figure 5C,D).

**Figure 6:**
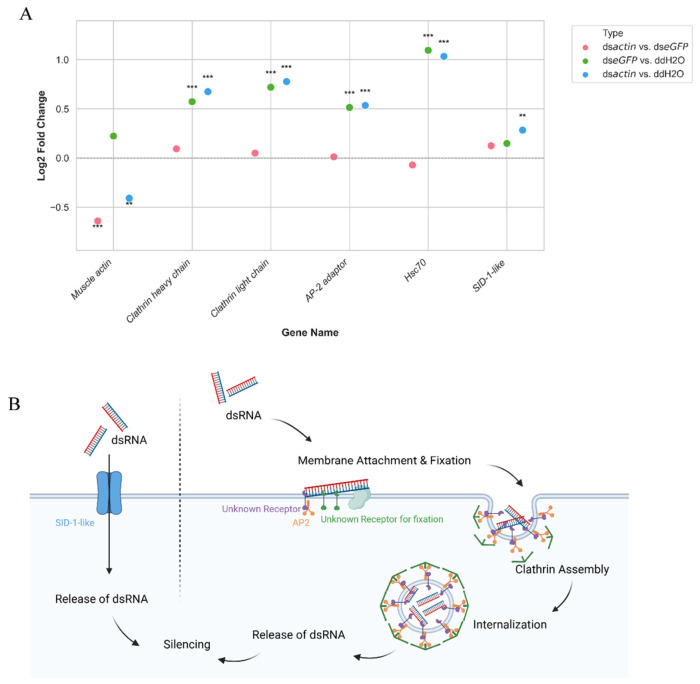
(**A**) Gene expression fold change across comparisons using transcriptome data. (**B**) Proposed model of dsRNA internalization in *M. usitatus* based on transcriptomic correlational data. Clathrin-mediated endocytosis and SID-1-like transmembrane transport are upregulated after dsRNA feeding. SID-1-like involvement is hypothetical and requires experimental validation. ** *p* < 0.01, *** *p* < 0.001.

Expression patterns of selected genes related to RNAi target, dsRNA transport, and cellular uptake are summarized in Figure 6A. The *muscle actin* gene showed a significant difference in the ds*actin* group compared with ds*eGFP* or the ddH_2_O control, indicating that exogenous ds*actin* significantly inhibited *muscle actin* gene expression in RNA-seq samples. The transcript levels of *SID-1-like*, *clathrin heavy chain*, *clathrin light chain*, *AP-2 adaptor*, and *Hsc70* were higher in both the ds*actin* and ds*eGFP* treatments than in the ddH_2_O control. Transcript levels of genes associated with clathrin-mediated endocytosis and SID-1-like transport were elevated following dsRNA exposure. A schematic overview of the proposed model based on these transcriptomic correlations is presented in Figure 6B.

### 3.5. Validation of RNA-seq Data by RT-qPCR

**Figure 7:**
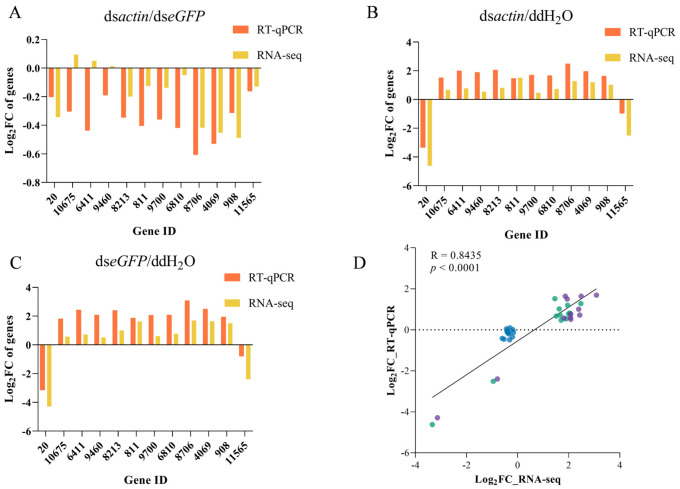
Validation of gene expression and correlations between RT-qPCR and RNA-seq data. (**A**–**C**) Bar charts showing the log_2_ fold changes in selected genes across three pairwise comparisons: ds*actin*/ds*eGFP* (**A**), ds*actin*/ddH_2_O (**B**), and ds*eGFP*/ddH_2_O (**C**). (**D**) Scatter plots illustrating the correlation of log_2_^Fold Changes^ between RNA-seq and RT-qPCR. Data points are color-coded by comparison group: blue = ds*actin*/ds*eGFP*, green = ds*actin*/ddH_2_O, purple = ds*eGFP*/ddH_2_O. Spearman correlation coefficients (R) and *p*-values (R = 0.8435, *p* < 0.0001) are shown.

To assess the reliability of the transcriptomic analysis, twelve genes representing diverse functional categories were selected for independent validation using RT-qPCR. The relative expression patterns obtained by RT-qPCR were consistent with those from RNA-seq across all three treatment comparisons: ds*actin* versus ds*eGFP*, ds*actin* versus ddH_2_O, and ds*eGFP* versus ddH_2_O (Figure 7A–C).

Correlation analysis between the log_2_^Fold Changes^ of genes derived from RNA-seq and RT-qPCR results showed a strong positive relationship (R = 0.8435, *p* < 0.0001; Figure 7D). These findings confirm the reproducibility of the expression data generated from the transcriptome sequencing.

## 4. Discussion

In this study, we established an optimized dsRNA production system in *E. coli* by engineering a vector containing three tandem terminators, which substantially increased dsRNA yield by approximately 11-fold compared to the terminator-free control. This enhancement likely results from improved transcription termination efficiency, reducing read-through transcription and ensuring the production of full-length dsRNA transcripts. Using this optimized platform, we provide molecular and physiological evidence that *muscle actin*-targeted dsRNA can induce mortality in two major thrips pests, *M. usitatus* and *F. occidentalis*, and that both clathrin-mediated endocytosis and SID-1-like membrane transport were transcriptionally upregulated following dsRNA exposure, suggesting their potential involvement in dsRNA uptake in Thysanoptera.

### 4.1. The Three-Terminator System: A Scalable Platform for High-Yield dsRNA Production

A substantial (~11-fold) enhancement of dsRNA yield was achieved using a pET28a-derived vector harboring three tandem transcriptional terminators (3 Ter: tT7 + rrnB T1 + tT7). This improvement likely results from synergistic termination efficiency, which minimizes transcriptional read-through—a common limitation of T7 RNAP that leads to heterogeneous transcripts and reduced yields of full-length dsRNA [9,38]. The observed growth retardation in the 3 Ter group suggests a trade-off: the metabolic burden of high-level dsRNA production temporarily slows bacterial proliferation but ultimately increases per-cell yield. From a translational perspective, this *E. coli*-based system offers a cost-effective alternative to in vitro synthesis.

The *E. coli* HT115(DE3) strain has been widely used for dsRNA production. In addition to the pET28a-derived vector developed in this study, other commonly used platforms include the L4440 and pLitmus28i vectors [39]. The L4440 vector, which consisted of two opposing convergent T7 promoters without transcriptional terminators, was first used by Timmons et al. to express dsRNA for RNAi in *C. elegans* [40]. More recently, the pLitmus28i system was reported to yield approximately 20 mg/L of dsRNA [41]. In our system, the yield produced by the pET28a vector containing three tandem terminators was significantly increased, as determined by Nanodrop quantification. However, a direct comparison of these vector systems under identical conditions is necessary to rigorously evaluate their relative productivity, ideally using mass spectrometry for absolute quantification in future studies.

### 4.2. Sequence Conservation and RNAi Efficacy of Muscle Actin in the Tested Thrips Species

Actin, a key cytoskeletal protein, plays an essential role in maintaining membrane curvature and coordinating with coat and scission proteins during cellular processes [42]. In insects, the major *actin* isoforms include *muscle actin* and *β-actin. Muscle actin* has rarely been investigated as a target for RNAi-based pest control. The *β-actin* gene has been successfully exploited as an RNAi target for pest control in several insect orders, including Thysanoptera [18], Hemiptera [43,44], and Coleoptera [45,46]. Despite the extensive application of *β-actin* in RNAi research attributed to its pleiotropic functions in core cellular pathways, our data reveal that *muscle actin*, which is specifically expressed in muscle, also confers robust insecticidal activity against thrips upon dsRNA ingestion. Notably, studies on the Colorado potato beetle (CPB, *Leptinotarsa decemlineata*) have shown that a sequence mismatch rate of <3% between the target gene and insecticidal dsRNA did not affect RNAi efficiency [47,48]. In line with this observation, our designed 400 bp *muscle actin* fragment shared >97% nucleotide identity between *M. usitatus* and *F. occidentalis*, ensuring sequence complementarity sufficient to elicit RNAi in both species.

Comparable knockdown efficiencies of *muscle actin* transcripts were detected in *M. usitatus* and *F. occidentalis*, yet the two species displayed divergent mortality phenotypes following dsRNA treatments. This discrepancy implies post-transcriptional regulatory mechanisms, such as protein turnover rate, are likely contributing determinants. Consistently, protein turnover rates are known to vary substantially across different insect species [49], and the stability of target proteins has been shown to significantly impact RNAi outcomes [50,51]. Rogers et al. [52] used RNAi to achieve 98% knockdown of *pro-resilin* mRNA, yet the phenotypic reduction in resilin was only 30 to 44%, which was considered to be affected by disparities between mRNA and protein expression levels. Conversely, in *Homalodisca vitripennis,* an 80% reduction in *actin* mRNA levels was observed by 5 days using RNAi, while a dramatic drop in actin protein levels occurred as early as 3 days [53]. Thus, we hypothesize that slower turnover of muscle actin in *F. occidentalis* prolongs the half-life of existing protein and consequently delays the onset of lethal RNAi effects. Future studies using Western blotting to measure muscle actin protein levels at different time points will directly test this hypothesis.

In this study, feeding bioassays demonstrated concentration-dependent mortality within 72 h of exposure, accompanied by significant suppression of *muscle actin* transcript levels. These results are consistent with previous reports of lethal *β*-act*in* silencing in *F. occidentalis* using transgenic tobacco expressing dsRNA [18]. Importantly, the present study achieved comparable RNAi efficacy via non-transgenic oral delivery—a more practical approach for field application. Furthermore, given that Thysanoptera and Hemiptera are considered sister orders based on genomic relationships and share similar sucking mouthparts [54], it is noteworthy that RNAi targeting *muscle actin* in the hemipteran *Euscelidius variegatus* also exhibited dose-dependent efficacy upon microinjection [43]. Collectively, these findings demonstrate that the highly conserved *muscle actin* fragment is an effective RNAi target in the two thrips species tested.

### 4.3. Transcriptomic Insights into dsRNA Uptake

RNAi efficiency in insects is primarily determined by dsRNA internalization, intracellular trafficking, and intercellular transport. In *M. usitatus*, transcriptome profiling following ds*actin* exposure revealed enrichment of pathways associated with protein digestion, lysosomal activity, and endocytosis, suggesting active engagement of cellular transport processes. The concurrent up-regulation of *clathrin heavy chain*, *clathrin light chain*, and *AP-2 adaptor* genes indicates transcriptional association with clathrin-mediated endocytosis (CME), whereas the elevated expression of *SID-1-like* transcripts points to the potential participation of transmembrane dsRNA transport channels. The coexistence of these transcriptional signatures parallels findings in *Leptinotarsa decemlineata* [24], where both CME and SID-1-like proteins cooperate to facilitate dsRNA uptake. In contrast, RNAi-refractory species such as *Drosophila melanogaster* lack functional SID-1 orthologs [55].

The transcriptomic data obtained in this study validate our prior hypotheses and enable the establishment of a proposed mechanistic framework explaining exogenous dsRNA dissemination in thrips [1]. As in other insects, the initial internalization in midgut cells likely involves membrane-associated receptors that recognize dsRNA, a process potentially facilitated by chaperones such as Hsc70 [56]. AP-2 [42], a key initiator of CME, is responsible for recognizing specific cargo motifs and recruiting clathrin to the plasma membrane. This leads to the assembly of clathrin heavy and light chains into a coated pit, which invaginates to form a vesicle, internalizing the receptor-dsRNA complex. Following endocytosis, the acidification of endosomal compartments via proton pumps is crucial for the release of dsRNA into the cytosol, where it can engage the core RNAi machinery, thereby completing the internalization and intracellular trafficking steps.

Although the function of SID-1 in mediating intercellular dsRNA transport is well established in *C. elegans* [57], the role of the upregulated SID-1-like homolog in *M. usitatus* remains hypothetical and requires direct experimental validation. Therefore, we propose a testable dual-pathway model wherein both CME and SID-1-like proteins contribute to dsRNA internalization, but definitive assignment of their respective roles requires targeted knockdown and pharmacological inhibition studies.

Beyond defining the uptake pathways, the expression data for CME and *SID-1-like* genes established in this study may serve as critical baselines for future resistance monitoring. Empirical evidence from multiple insect species supports the involvement of these internalization pathways in RNAi resistance. In the Colorado potato beetle, resistance to dsRNA is associated with suppressed clathrin-mediated endocytosis and reduced expression of the *clathrin heavy chain* gene [58]. Similarly, in the western corn rootworm, impaired luminal uptake of dsRNA, rather than enhanced degradation, has been identified as a primary resistance mechanism [59]. Furthermore, reduced dsRNA uptake is recognized as a key barrier to RNAi in insects, and alterations in endocytic pathway genes have been linked to resistance development [8,60]. Given these findings, the baseline expression profiles for CME and SID-1-like pathway genes in *M. usitatus* provide a direct reference point.

Exogenous dsRNA alone can trigger a general, non-specific transcriptional response in insects, independent of its sequence. This phenomenon is well-documented in the order Hymenoptera, including *Nasonia vitripennis* and *Apis mellifera*, where *GFP* dsRNA treatment has been shown to alter the expression of hundreds of genes involved in immunity, stress, and metabolism [61,62]. A similar non-specific transcriptional response was observed in our study (Figure 5B). For instance, a slight, non-significant upregulation of *muscle actin* transcripts was detected in the ds*eGFP* control group (Figure 6A), reflecting a background stress response. Critically, this non-specific effect remained marginal and did not translate into any observable phenotypic consequences. In contrast, ds*actin* treatment resulted in a significant, robust knockdown of *muscle actin* transcripts, and a strong correlation between target gene silencing and mortality. These findings demonstrate that while a general dsRNA response exists, the observed lethality is unequivocally driven by the sequence-specific silencing of *muscle actin*, and not solely by the general dsRNA response.

### 4.4. Implications for RNAi-Based Biopesticide Design

The commercial viability of non-transgenic RNAi-based biopesticides as an additional tool for IPM strategies has been demonstrated. GreenLight Biosciences obtained US Environmental Protection Agency (EPA) registration for two insecticides (Calantha targeting CPB and Norroa targeting *Varroa destructor*), while Silicon Gene Technologies obtained registration from the Ministry of Agriculture and Rural Affairs of China for one fungicide (Tomovircona targeting tobacco mosaic virus) [63].

Building on this commercial progress, the present study strengthens the foundation for practical RNAi-based pest control by integrating an optimized dsRNA production system with transcriptomic insights into dsRNA uptake. However, whether the *muscle actin* target identified in this study can be further developed into a broadly applicable RNAi-based biopesticide depends on several factors. First, its lethal efficacy must be validated across additional thrips species to evaluate species-specific differences. Second, off-target effects on natural enemies, beneficial insects, or other non-target organisms must be systematically assessed. Computational predictions can guide these evaluations and then be confirmed through designed bioassays.

The identification of dual uptake routes has practical implications for improving dsRNA delivery formulations. CME is energy-dependent and can be enhanced by nanoparticles or carrier complexes that promote vesicular uptake [64], whereas SID-1-like transport relies on SID-1-like-mediated dsRNA internalization across membranes. Incorporating these transcriptomic insights into formulation design—such as star polymer [64], liposomes [65], or layered double hydroxide (LDH) [66]—may increase stability and uptake efficiency under field conditions. Because environmental degradation of naked dsRNA occurs rapidly through UV exposure and nuclease activity [1,8], protecting dsRNA while maintaining bioavailability will be essential for effective thrips control. The *muscle actin*-based dsRNA characterized in this study can serve as a model molecule to test carrier performance and delivery optimization.

### 4.5. Broader Significance and Future Research

A conserved *muscle actin* fragment induces RNAi in two distinct thrips species, highlighting a key strategic consideration for RNAi-based biopesticide development: the trade-off between species-specific and conserved target genes.

Species-specific targets may offer enhanced selectivity but require the development and regulatory approval of multiple products for pest complexes. In contrast, targeting evolutionarily conserved essential genes, as demonstrated here, enables simultaneous suppression of co-occurring thrips species such as *M. usitatus* and *F. occidentalis* using a single dsRNA active ingredient. This “multi-species” targeting strategy offers economic and practical advantages but necessitates rigorous non-target risk assessment. Future research should systematically evaluate the efficacy of this ds*actin* across additional Thysanoptera species and conduct toxicological assays on representative non-target arthropods (e.g., pollinators and predatory insects) and natural enemies to define the ecological boundaries.

Although transcriptomic evidence suggests the involvement of CME- and SID-1-like-mediated dsRNA transport, definitive functional validation is still required. Targeted silencing of *clathrin heavy chain* or *SID-1-like* genes, as well as pharmacological inhibition of dsRNA internalization, could elucidate their individual and relative contributions to dsRNA uptake. Expanding these analyses across developmental stages will further determine whether this dual uptake mechanism is conserved within Thysanoptera. Integrating such transcriptomic insights with formulation research will accelerate the translation of RNAi from laboratory studies to field-applicable pest management solutions.

## 5. Conclusions

This study demonstrates that *muscle actin*-targeted double-stranded RNA (dsRNA) can effectively induce RNA interference (RNAi) and cause mortality in both *Megalurothrips usitatus* and *Frankliniella occidentalis*, two of the most economically significant thrips pests.

In this study, we began by establishing an optimized dsRNA production system in *E. coli* by engineering a vector containing three tandem terminators, which substantially increased dsRNA yield and provided sufficient high-quality dsRNA for subsequent mechanistic and bioassay studies. Using this optimized platform, we demonstrated that *muscle actin*-targeted dsRNA can effectively trigger RNAi responses and induce significant mortality in two agriculturally important thrips pests, *M. usitatus* and *F. occidentalis*. The selected *muscle actin* fragment is highly conserved across the two tested thrips species, and oral delivery of dsRNA targeting this region resulted in significant gene silencing and mortality. These findings support *muscle actin* as a promising RNAi target for thrips control. However, cross-species potential requires empirical validation.

Transcriptome analysis revealed that dsRNA uptake in *M. usitatus* may be associated with two molecular pathways: clathrin-mediated endocytosis (CME) and SID-1-like transmembrane transport. The co-activation of these mechanisms provides the first evidence for dual dsRNA internalization routes in thrips. This transcriptomic insight advances the current understanding of how exogenous RNA molecules are absorbed and processed in RNAi-sensitive insects. The transcriptomic correlations observed here suggest a potential dual-pathway model for dsRNA uptake. Knowledge of CME and SID-1-like involvement can inform the design of formulation additives or nanocarriers that promote cellular entry and protect dsRNA from degradation. Such integration of molecular biology and formulation science will be essential for translating RNAi from laboratory success to field-ready biopesticides.

Future research should functionally characterize the specific roles of *clathrin heavy chain* and *SID-1-like* genes through targeted knockdown and cellular imaging. Expanding these analyses across developmental stages and additional Thysanoptera species will clarify the evolutionary consistency of this uptake mechanism. Collectively, these findings establish a proposed mechanistic framework for dsRNA uptake in Thysanoptera that is likely relevant to other rasping-sucking and piercing-sucking insects, thereby broadening the applicability of RNAi-based pest management strategies beyond thrips and supporting the rational design of RNAi-based biopesticides for a wider range of economically important pests.

## Data Availability

The transcriptome sequencing data from this study are publicly available in the NCBI Sequence Read Archive (SRA) under BioProject accession number PRJNA1397900. The associated BioSample and SRA run accessions are SAMN54436043: dsactin and SRR36668365, SAMN54436044: dseGFP and SRR36668366, SAMN54436045: ddH2O and SRR36668367, respectively.

## Supplementary Materials

The following supporting information can be downloaded at: https://www.mdpi.com/article/10.3390/insects17070685/s1, Figure S1: Stability of dsRNA targeting *muscle actin* in artificial diet at concentrations of (A) 300 ng μL^−1^ and (B) 1500 ng μL^−1^. M: 2000 bp plus DNA ladder; h: hours. A total of 300 ng dsRNA and 5 μL of DNA marker were loaded per lane; Figure S2: Sequence similarity between dsRNA targeting *muscle actin* (ds*actin*), *muscle actin* in *M. usitatus*, and *muscle actin* in *F. occidentalis*; Table S1: Details of primers used in this study; Table S2: Sequences of dsRNAs/Oligonucleotide used in this study; Table S3: GenBank accession numbers of the *muscle actin* gene used to construct a phylogenetic tree in Figure 2. Outgroup: *Frankliniella occidentalis* (XM_026437356.2), used as a control; Supplementary Methods: Plasmid construction details.
