# Supplementary material for: Enhanced dsRNA Production via a Three-Terminator Vector and Transcriptomic Correlates of RNAi Exposure in Thrips
Source: Insects. 2026 Jul 1;17(7):685. doi: 10.3390/insects17070685 (PMC13410476; doi:10.3390/insects17070685)
Supplement: Supplementary file 1 [file insects-17-00685-s001.zip › insects-4335260-supplementary.pdf]

Supplementary material

Enhanced dsRNA Production via a Three-Terminator Vector and Transcriptomic Correlates of RNAi Exposure in Thrips

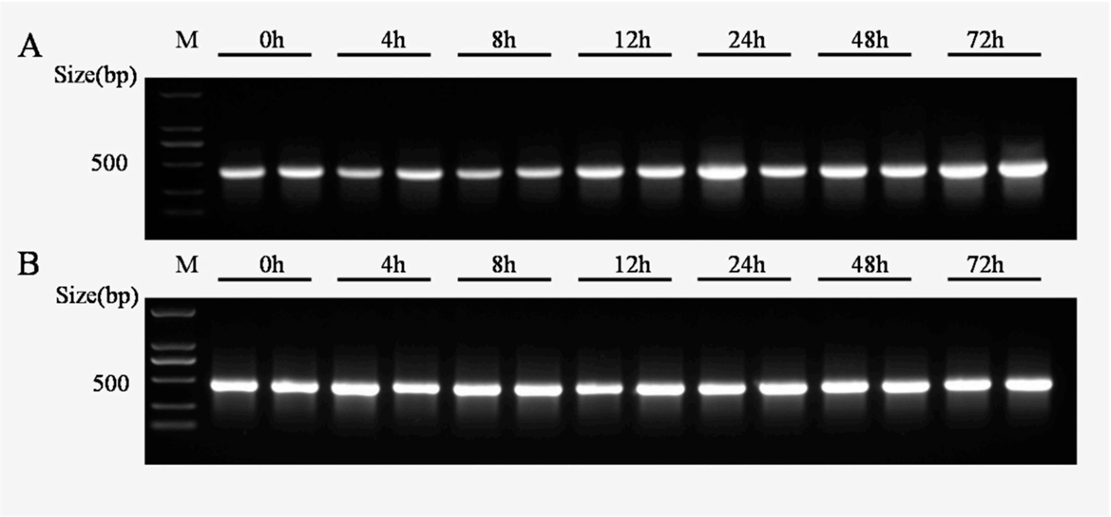

Figure S1. Stability of dsRNA targeting *muscle actin* in artificial diet at concentrations of (A) 300 ng μL^{-1} and (B) 1500 ng μL^{-1}. M: 2000 bp plus DNA ladder; h: hours. A total of 300 ng dsRNA and 5μL of DNA marker was loaded per lane.

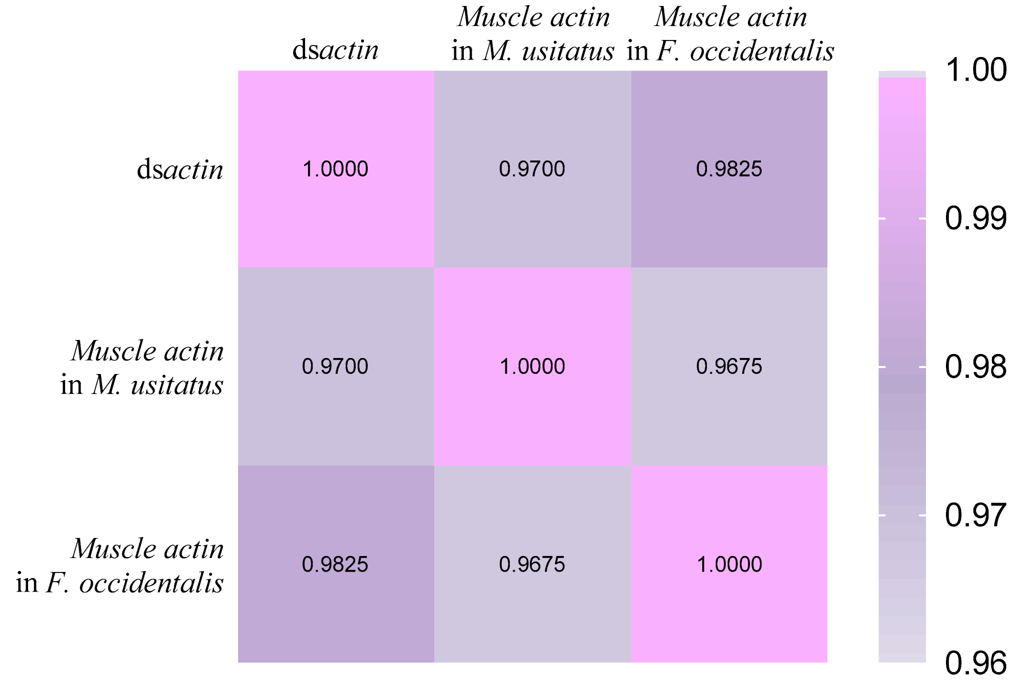

Figure S2. Sequence similarity between dsRNA targeting *muscle actin* (ds*actin*), *muscle actin* in *M. usitatus*, and *muscle actin* in *F. occidentalis*.

Table S1. Details of primers used in this study.

No.	Primer name	Gene name	Primer sequence (5'-3')	Product length	Annealing temperature in (q)PCR	Purpose	References
1	LCOI1490<br>HC2198	*mtCOI*	GGTCAACAAATCATAAAGATATTGG<br>TAAACTTCAGGGTGACCAAAAAATCA	710bp	55℃	identification of *M. usitatus*	[31]
2	CAS5p8sFc<br>CAS28sB1d	*mtCOI*	TGAACATCGACATTTYGAACGCACAT<br>TTCTTTTCCTCCSCTTAYTRATATGCTTAA	490bp	55℃	identification of *F. occidentalis*	[32]
3	actin	*Muscle actin*	ACGCCATCCTCCGTCTGGACT<br>TACATGGTGGTACCACCGGACA	400bp	60℃	dsRNA synthesis	this study
4	actin	*Muscle actin*	CACCGCTGAGCGTGAAATCG<br>TGACCTGACCGTCGGGAAGC	137bp	60℃	RT-qPCR as the target gene of *M. usitatus*	this study
5	actin	*Muscle actin*	GGACTTCGAGCAGGAAATGG<br>TGAAGGCTGGAACAGGGACT	133bp	60℃	RT-qPCR as the target gene of *F. occidentalis*	this study
6	gapdh	*gapdh*	ACTGTTGATGGTCCCTCTGG<br>AGCGGCTTCCTTAACCTTCT		60℃	RT-qPCR as a housekeeping gene of *M. usitatus*	[35]
7	tubulin	*tubulin*	TTCCACTGCTGTTGTTGAGC<br>AGATGGCCTCATTGTCAACC		60℃	RT-qPCR as a housekeeping gene of *F. occidentalis*	[36]
8	20	*lysosomal aspartic protease-like isoform X2*	GCTGGTTCTGGAACATTGCTT<br>GGAGACGGAGTCGGTTGAGTA	161bp	60℃	RT-qPCR in *M. usitatus* for transcriptome validation	this study
9	10675	*clathrin heavy chain*	AGCAGAGCACGATCACAAACCC<br>ACCAGGTGCCGCTGTATAACCA	121bp	60℃	RT-qPCR in *M. usitatus* for transcriptome validation	this study

10	6411	*clathrin light chain isoform X1*	GGAGATCAAGACCCGTTTGCAAG<br>GGGCGCATCACCAAGACTAATTT	210bp	60℃	RT-qPCR in *M. usitatus* for transcriptome validation	this study
11	9460	*AP-2 complex subunit alpha*	TGACTGCCGCTACATCACTTAT<br>AACAGCTTGACAGACAGCCAA	252bp	60℃	RT-qPCR in *M. usitatus* for transcriptome validation	this study
12	8213	*RISC-loading complex subunit tarbp2-like*	ACATGAGCCTACATTCCGCTAC<br>TGTCCACCCATCAACTTATCCAG	126bp	60℃	RT-qPCR in *M. usitatus* for transcriptome validation	this study
13	811	*charged multivesicular body protein 6*	GGGAGCACTCTTTGGAAAGCAG<br>CACGCAGAAGTTTACGAGCCA	161bp	60℃	RT-qPCR in *M. usitatus* for transcriptome validation	this study
14	9700	*ABC transporter G family member 20 isoform X1*	TCCTTCCGGTTGTGATGCTT<br>AGCCTCTTGCCATCATCGCT	132bp	60℃	RT-qPCR in *M. usitatus* for transcriptome validation	this study
15	6810	*exocyst complex component 3*	GCTGCTTACTTTGAGGATGTGGA<br>AAGGGCTGTGACTATGATTGTGG	120bp	60℃	RT-qPCR in *M. usitatus* for transcriptome validation	this study
16	8706	*hypothetical protein FOCC_FOCC014660*	TGAAGGCGTGTCTGAAGCTG<br>ATTGTTTGAAGCTAAGCCAGGG	116bp	60℃	RT-qPCR in *M. usitatus* for transcriptome validation	this study
17	4069	*ATP-dependent helicase brm-like isoform X2*	ATCAAGATGAGGAAGAAAACGAGG<br>TCGCTTCTTCACGTCGTCTTTC	123bp	60℃	RT-qPCR in *M. usitatus* for transcriptome validation	this study
18	908	*GILT-like protein 1*	AATCCCAGTACACAAGGGCTGA<br>ACAGTTGAGGTAGGAGAGACGT	143bp	60℃	RT-qPCR in *M. usitatus* for transcriptome validation	this study
19	11565	*heat shock 70 kDa protein-like isoform X2*	ACAATGGACACCCGAAAGTGC<br>TCTGGCTGTCCGTGAAGTAGGC	144bp	60℃	RT-qPCR in *M. usitatus* for transcriptome validation	this study

Table S2. Sequences of dsRNAs/Oligonucleotide used in this study.

dsRNAs/Oligonucleotide	Sequence (5'→3')
ds*actin*	ACGCCATCCTCCGTCTGGACTTGGCTGGTCGCGATCTGACTGACTACCTCATGAAGATCCTCACCGAGCGTGGTTACTCTTTCGTCACCACCGCTGAGCGTGAAATCGTTCGTGACATCAAGGAGAAGCTCTGCTACGTCGCCCTGGACTTCGAGCAGGAAATGGCCACCGCCGCCGCCTCCACCTCCCTCGAGAAGTCCTACGAGCTTCCCGACGGTCAGGTCATCACCATCGGTAACGAGAGGTTCCGCGCTCCCGAGTCCCTGTTCCAGCCTTCATTCGTGGGCATGGAATCTTGCGGTATCCACGAGACCGTCTACAACTCCATCATGAAGTGCGACGTCGACATCCGTAAGGACCTGTACGCCAACAATGTCCTGTCCGGTGGTACCACCATGTA
ds*eGFP*	GAAGCAGCACGACTTCTTCAAGTCCGCCATGCCCGAAGGCTACGTCCAGGAGCGCACCATCTTCTTCAAGGACGACGGCAACTACAAGACCCGCGCCGAGGTGAAGTTCGAGGGCGACACCCTGGTGAACCGCATCGAGCTGAAGGGCATCGACTTCAAGGAGGACGGCAACATCCTGGGGCACAAGCTGGAGTACAACTACAACAGCCACAACGTCTATATCATGGCCGACAAGCAGAAGAACGGCATCAAGGTGAACTTCAAGATCCGCCACAACATCGAGGACGGCAGCGTGCAGCTCGCCGACCACTACCAGCAGAACACCCCCATCGGCGACGGCCCCGTGCTGCTGCCCGACAACCACTACCTGAGCACCCAGTCCGCCCTGAGCAAAGACCCCAACGAGAAGCGCGATCACATGGTCCTGCTGGAGTTC
tT7	CAAAAAACCCCGCGAGACCCCTTTCAGCCGAAGAGGCCCCGCGGGGTT
rrnB T1	ATAAAACGAAAGGCCCAGTCTTCTTTCAGTCGACTGAGCCTTTCGTTTTATTTG

Table S3. GenBank accession numbers of *muscle actin* gene used to construct a phylogenetic tree in Fig. 2. Outgroup: *Frankliniella occidentalis* (XM_026437356.2), used as control.

	Organism name	GenBank accession number	Order
1	*Megalurothrips usitatus*	OR711914.1	Thysanoptera
2	*Frankliniella occidentalis*	XM_052268144.1	Thysanoptera
3	*Thrips palmi*	XM_034388025.1	Thysanoptera
4	*Macrosteles quadrilineatus*	XM_054409806	Hemiptera
5	*Oncometopia nigricans*	AY725778.1	Hemiptera
6	*Nilaparvata lugens*	XM_022345417.2	Hemiptera
7	*Euscelidius variegatus*	GFTU01006510.1	Hemiptera
8	*Papilio machaon*	XM_014502564	Lepidoptera
9	*Papilio polytes*	AK402249	Lepidoptera
10	*Epargyreus clarus*	XM_073090209	Lepidoptera
11	*Manduca sexta*	XM_030172658.2	Lepidoptera
12	*Plutella xylostella*	AB282645.1	Lepidoptera
13	*Melitaea cinxia*	XM_045599032.1	Lepidoptera
14	*Neodiprion fabricii*	XM_046577382	Hymenoptera
15	*Athalia rosae*	XM_012408090	Hymenoptera
16	*Neodiprion virginianus*	XM_046765063	Hymenoptera
17	*Neodiprion pinetum*	XM_046623590	Hymenoptera
18	*Neodiprion lecontei*	XM_015660053	Hymenoptera
19	*Cephus cinctus*	XM_025090279	Hymenoptera
20	*Schistocerca piceifrons*	XM_047246322	Orthoptera
21	*Schistocerca americana*	XM_047128817.1	Orthoptera
22	*Schistocerca cancellata*	XM_049926815.1	Orthoptera
23	*Schistocerca nitens*	XM_049950248.1	Orthoptera
24	*Schistocerca gregaria*	XM_049991084.1	Orthoptera

Supplementary Methods: Plasmid construction details

Construction of the 3Ter plasmid (pET28a-3Ter-cloning region-3Ter). The commercial pET28a vector was digested with SphI (4775) and XbaI (5030). A synthetic DNA fragment (Genewiz, Azenta Life Sciences) containing one T7 promoter and three tandem T7 terminators, flanked by homology arms, was assembled into the linearized vector via homologous recombination (Gibson assembly). The resulting intermediate plasmid, pET28a-3Ter, contained one T7 promoter and three tandem terminators on one side. This intermediate was then digested with XbaI (5020) and HindIII (5182). A second synthetic DNA fragment (Genewiz) containing one T7 promoter, three T7 terminators, and the cloning region was also digested with XbaI and HindIII. The digested fragment was ligated into the linearized intermediate vector, yielding the final 3Ter plasmid, which contains two convergent T7 promoters, the cloning region, and three terminators on each side.

Construction of the 1Ter plasmid (pET28a-1Ter-cloning region-1Ter). The 3Ter plasmid was digested with SphI (4775) and XbaI (5020). A synthetic DNA fragment (Genewiz) containing one T7 promoter and one T7 terminator, flanked by homology arms, was assembled into the linearized vector via homologous recombination. This produced an intermediate plasmid, pET28a-1Ter-cloning region-3Ter, containing one T7 promoter and one terminator on one side, and three terminators on the other. This intermediate was then digested with SacI (5335) and HindIII (5528). A second synthetic DNA fragment (Genewiz) containing one T7 promoter and one T7 terminator, flanked by homology arms, was assembled into the linearized intermediate via homologous recombination, yielding the final 1Ter plasmid.

Construction of the 0Ter plasmid (pET28a-0Ter-cloning region-0Ter). The 1Ter plasmid was digested with SphI (4775) and XbaI (4875). A synthetic DNA fragment (Genewiz) containing one T7 promoter was digested with the same restriction enzymes and ligated into the linearized vector. This produced an intermediate plasmid, pET28a-0Ter-cloning region-1Ter. This intermediate was then digested with KpnI (5223) and HindIII (5360). A second synthetic DNA fragment (Genewiz) containing one T7 promoter was digested with the same restriction enzymes and ligated into the linearized intermediate, yielding the final 0Ter plasmid, which contains two convergent T7 promoters and the cloning region without any terminators.
